# Anti-Tumor Effects of Peptide Therapeutic and Peptide Vaccine Antibody Co-targeting HER-1 and HER-2 in Esophageal Cancer (EC) and HER-1 and IGF-1R in Triple-Negative Breast Cancer (TNBC)

**DOI:** 10.3390/vaccines3030519

**Published:** 2015-07-06

**Authors:** Jay Overholser, Kristen Henkins Ambegaokar, Siobhan M. Eze, Eduardo Sanabria-Figueroa, Rita Nahta, Tanios Bekaii-Saab, Pravin T.P. Kaumaya

**Affiliations:** 1 Department of Obstetrics and Gynecology, The Ohio State University Wexner Medical Center, Columbus, OH 43210, USA; E-Mails: jay.overholser@osumc.edu (J.O.); kristen.ambegaokar@osumc.edu (K.H.A.); 2 Department of Pharmacology, Emory University and Winship Cancer Institute, Atlanta, GA 30322, USA; E-Mails: siobhan.maureen.donnelly@emory.edu (S.M.E.); rnahta@emory.edu (R.N.); 3 Molecular and Systems Pharmacology Program, Graduate Division of Biological and Biomedical Sciences, Emory University, Atlanta, GA 30322, USA; E-Mail: esanab2@emory.edu; 4 James Cancer Hospital and Solove Research Institute and the Comprehensive Cancer Center, The Ohio State University, Columbus, OH 43210, USA; E-Mail: Tanios.Saab@osumc.edu

**Keywords:** peptide mimics, epitopes, antibodies, immunogenicity, resistance, vaccine candidates, peptide vaccines

## Abstract

Despite the promise of targeted therapies, there remains an urgent need for effective treatment for esophageal cancer (EC) and triple-negative breast cancer (TNBC). Current FDA-approved drugs have significant problems of toxicity, safety, selectivity, efficacy and development of resistance. In this manuscript, we demonstrate that rationally designed peptide vaccines/mimics are a viable therapeutic strategy for blocking aberrant molecular signaling pathways with high affinity, specificity, potency and safety. Specifically, we postulate that novel combination treatments targeting members of the EGFR family and IGF-1R will yield significant anti-tumor effects in *in vitro* models of EC and TNBC possibly overcoming mechanisms of resistance. We show that the combination of HER-1 and HER-2 or HER-1 and IGF-1R peptide mimics/vaccine antibodies exhibited enhanced antitumor properties with significant inhibition of tumorigenesis in OE19 EC and MDA-MB-231 TNBC cell lines. Our work elucidates the mechanisms of HER-1/IGF-1R and HER-1/HER-2 signaling in these cancer cell lines, and the promising results support the rationale for dual targeting with HER-1 and HER-2 or IGF-1R as an improved treatment regimen for advanced therapy tailored to difference types of cancer.

## 1. Introduction

Gastric and gastroesophageal cancers remain the sixth most common cause of cancer deaths worldwide [1,2] with an increasingly high mortality rate, as the disease is generally diagnosed at an advanced stage. Esophageal tumor types are typically very aggressive, and effective treatments, particularly targeted therapies available, remain elusive [3]. Adenocarcinoma remains the leading type of esophageal cancer (EC) and increased rates of these cancers are seen in patients with Barrett’s esophagus and esophageal reflux disease [4,5]. The survival rates of patients within this group of diseases remain extremely low, typically less than one year with therapy [6]. Triple-negative breast cancer (TNBC) accounts for 12%–24% of all breast cancer cases and is one of the most aggressive subtypes known [7]. TNBCs exhibit higher rates of relapse during early stages and decreased overall survival in the metastatic setting. TNBC affects younger patients and African-American women more frequently that other patient populations. In fact, TNBCs account for about 30% of all breast cancers diagnosed in African-American females [8]. TNBC is characterized by a triple-negative receptor status, with loss of estrogen receptor (ER), progesterone receptor (PR) and HER-2. Thus, TNBC remains unresponsive to hormonal therapy and HER-2 targeted antibody therapy [7,9]. Therefore, cytotoxic chemotherapy remains the main treatment for patients with TNBC. Although some patients respond, the treatment is toxic, and a large percentage of patients who are treated in the early stages of disease eventually relapse within five years. Relapse after chemotherapy confers a dismal prognosis, and patients eventually succumb to their disease. Thus, there is an urgent need to develop novel, safe, and effective therapeutic strategies for EC and TNBC.

Significant advances in our understanding of the signaling networks that drive cancer progression have ushered in a new era of cancer therapeutics. These new agents inhibit specific growth stimulatory pathways, including receptor tyrosine kinase (RTK) cascades. The epidermal growth factor receptors (HER-1, HER-2, HER-3 and HER-4), vascular endothelial growth factor receptor (VEGFR) [10,11,12,13,14,15,16,17,18,19,20,21], and insulin-like-growth factor receptor-1 (IGF-1R) [22,23] are among the most well-studied RTKs. A plethora of FDA-approved agents targeted against RTK signaling pathways [24,25,26] are directed against HER-2 (trastuzumab, pertuzumab, lapatinib), EGFR (cetuximab, gefitinib, erlotonib) or VEGF (bevacizumab, sunitinib). These agents have markedly improved survival but demonstrate significant toxicities [27,28,29]. Furthermore, clinical applications of humanized monoclonal antibody (hmAb) therapy are limited by a number of concerns such as the high frequency of treatments, associated costs, limited duration of action, undesired immunogenicity, and significant risk of cardiotoxicity. Similarly, while small-molecule RTK inhibitors, such as erlotinib and lapatinib, are approved as single agents or in combination with radiotherapy or chemotherapy, these inhibitors display limited and transient efficacy, development of resistance, and significant toxicities.

HER-2 is overexpressed in gastric (21%) or gastroesophageal junction cancers (33%), in association with an increased risk of metastasis, decreased survival [30], and increased tumor invasion [31]; this correlation between HER-2 overexpression and poor prognostic markers established HER-2 as a potential therapeutic target. Studies have shown the beneficial effects of the HER-2 hmAb trastuzumab, in cell lines [32] and orthotopic mouse models of HER-2 EC [33]. Based on data from the ToGA trial, trastuzumab was approved in 2010 for patients with HER-2-positive metastatic gastric adenocarcinoma in combination with chemotherapy [34,35]. Additionally, the HER-2 targeted hmAb pertuzumab has had significant clinical success in the treatment of breast cancer when combined with trastuzumab [36]; similar dual-targeting clinical trials are under way for EC (NCT02120911). HER-1 overexpression is also correlated with poor prognosis in EC [37] and is implicated in the progression of Barrett’s esophagus to adenocarcinoma [38]. The upregulation of HER-1 in EC makes it an attractive target for drug therapy. Several studies have reported the dual overexpression of HER-1 and HER-2 in EC with extremely high rates in esophageal adenocarcinoma when compared to that of squamous cell carcinomas of the esophagus [39,40,41,42]; thus, there is a potential clinical benefit in co-targeting HER-1 and HER-2 in EC [32,43]. HER-1 and HER-2 targeted inhibitors are widely considered a potential therapeutic strategy in the treatment of EC [44].

HER-1 is overexpressed in 70% of basal-like TNBCs [45,46] which correlates with poor prognosis [47] and low survival rates [48]; thus HER-1 is a putative therapeutic target [49] in TNBC. This observation led to the evaluation of the anti-HER-1 antibody cetuximab in the clinical setting as single and combination treatments [50,51]. HER-1-targeting with hmAb cetuximab performed poorly in metastatic breast cancer. However, further trials combining cetuximab with cisplatin showed significant success in phase II trials, establishing HER-1 as an important molecular target in TNBC [51]. A subset of TNBC cells also shows significant upregulation of IGF-1R, both at the gene and protein levels, suggesting that IGF-1R is also an important therapeutic target in TNBC. IGF-1R is highly implicated in TNBC, and therapies targeted against IGF-1R signaling inhibit the growth of TNBC cells [52]. Anti-IGF-1R therapies suppressed tumor growth and development in mouse xenografts of human TNBC, providing rationale for targeting IGF-1R alone or in combination with agents targeted against other receptors in TNBC [53]. High expression of IGF-1R correlates with metastatic disease [52]. Further, increased expression of IGF-1R can increase expression of HER-1 and result in the formation of IGF-1R/HER-1 dimers [54,55]. Thus, there is considerable evidence of cross-talk between HER-1 and IGF-1R in TNBC.

Therapeutic strategies that target single molecular pathways eventually succumb to problems of intrinsic or acquired resistance due to extensive signaling “crosstalk”. Thus, combination targeted therapies are more attractive, as they synergistically inhibit multiple receptors. Recently, we have developed novel strategies [56,57] to target multiple RTKs simultaneously. In TNBC, there is considerable evidence of cross-talk between HER-1 and IGF-1R [58], suggesting that combination therapy against these two receptors could yield better outcomes. Indeed, a preclinical study showed that co-inhibition of HER-1 and IGF-IR sensitized HER-1/IGF-1R expressing human breast cancer cells to radiation [59]. FDA-approved drugs targeting HER-1 (gefitinib) and IGF-1R (AG1024) respectively show synergistic therapeutic advantage in *in vitro* human breast cancer cells, further highlighting the importance of pursuing combination therapies in cancer treatment [60]. Combination therapy has also demonstrated clinical success in gastric cancers. Notably, lapatinib produced synergistic antitumor effects *in vitro* when combined with 5-fluorouracil in the treatment of EC [61]. Further, a large phase III trial (ToGA) testing the combination of chemotherapy with trastuzumab in HER-2 positive gastroesophageal cancers showed an increase in response rate and overall survival with combination treatment [35]. Taken together, these studies provide strong rationale for investigating novel combination strategies using inhibitors against HER-1, HER-2 and IGF-IR to treat patients with EC or TNBC.

Although combination therapy is a promising avenue of investigation, targeted and effective treatments for EC and TNBC remain to be found. Unfortunately, patients generally develop secondary resistance to monoclonal antibody regimens, such as trastuzumab [62]; the development of resistance may be exacerbated by tumor heterogenicity in EC [63,64]. Dual targeting with hmAbs is limited by the potential for overlapping and enhanced toxicity, prohibiting administration of the full established dose of either agent; thus, many clinical trials have yielded mixed results [65]. Phase II clinical trials with the HER-1 inhibitor gefitinib and the HER-2 hmAb trastuzumab failed to show a synergistic effect in patients with metastatic breast cancer [66]. Further, the combination of the HER-1 kinase inhibitor erlotinib and VEGF hmAb bevacizumab showed little therapeutic benefit in a phase II trial of renal cell cancer. However, the HER-1 hmAb cetuximab combined with the VEGF hmAb bevacizumab showed promising synergy in preliminary data obtained in colorectal cancer [67]. Clearly, an urgent need exists for novel combinations that can safely overcome resistance mechanisms. These agents must be rationally designed to fit the molecular profile of the specific tumor type being treated.

We hypothesized that the novel combination treatment of peptide mimics or peptide vaccine antibodies against HER-1 with HER-2, and HER-1 with IGF-1R will significantly inhibit tumorigenesis in *in vitro* models of EC and TNBC, respectively. Previously, we designed two novel HER-2 B-cell epitope peptide vaccines (HER-2-266-296, pertuzumab-like, and HER-2-597-629, trastuzumab-like) and demonstrated antitumor effects in several *in vitro* and *in vivo* models of human breast cancers [68,69]. A combination of these two peptide vaccines is undergoing an FDA-approved, NCI-funded phase 1 clinical trial (NCT01376505) at the Ohio State University James Cancer Hospital and the Comprehensive Cancer Center. We have also identified two novel HER-1 ligand-binding epitopes and have shown antitumor properties in both *in vitro* and *in vivo* models of breast and lung cancers [70]. In addition to our vaccine strategies, we have demonstrated that peptide mimics also represent a safe and viable therapeutic option for blocking aberrant signaling pathways with high affinity and strong potency. In previous publications, we showed that our HER-2 and VEGF peptide mimics [56,71] specifically target the HER-2 and VEGF pathway and do not exhibit off-target effects. Peptide mimics offer the benefits of being water-soluble, non-immunogenic, low in manufacturing cost, and having an enhanced shelf life with the ability to easily cross tissue barriers [72].

In this paper, we demonstrate that a novel combination approach using peptide mimics and peptide vaccine antibodies significantly inhibited cancer signaling pathways *in vitro*. Combination treatment with HER-1 and HER-2 in EC or HER-1 and IGF-1R in TNBC exhibited increased anti-tumor responses, including reduced proliferation, decreased receptor phosphorylation, increased antibody-dependent cellular cytotoxicity (ADCC) and increased apoptosis in EC (OE19) and TNBC (MDA-MB-231) cell lines. Ongoing and future *in vivo* studies in xenograft mouse models will further validate these *in vitro* results. Ultimately, these studies may lead to new therapeutic strategies for EC and TNBC.

## 2. Materials and Methods

### 2.1. Peptide Selection, Design and Peptide Synthesis

We have identified, designed, synthesized and tested novel peptide sequences that target HER-1, HER-2 and IGF-1R as previously described [68,69,70]. The HER-1 and IGF-1R sequences were derived from the ligand binding domains of the receptors and block ligand-induced signaling in cancer cells that express these receptors. The two HER-2 peptide epitopes were designed based on the crystallographic structures of the humanized monoclonal antibodies trastuzumab and pertuzumab in complex with HER-2. Peptide synthesis was performed using 9600 Milligen/Biosearch solid-phase peptide synthesizer (Millipore, Bedford, MA, USA) using Fmoc/t-Butyl chemistry and PyBOP/HOBT coupling reagents on either CLEAR amide resin or CLEAR acid resin (Peptides International, Louisville, KY, USA). All MVF derived chimeric peptide vaccines were co-linearly synthesized with a promiscuous Th cell epitope derived from the measles virus fusion protein (MVF; residues 288–302) using a four residue linker (GPSL). Peptide mimics were acetylated using 1-Acetylimidazole (Sigma-Aldrich St. Lois, MO, USA) before cleavage. Intramolecular disulfide bonds were formed using iodine oxidation and disulfide bridge formation was further confirmed by maleimide-PEO2-biotin reaction and subsequent analysis using electrospray ionization mass spectroscopy. Peptides were cleaved from the resin using cleavage reagent R (TFA)/thioanisole/EDT/anisole (90/5/3/2), and crude peptides were purified by semi preparative (C-4 or C-18 Vydac columns) reversed-phase-HPLC (Waters, Bedford, MA, USA) and characterized by MALDI (Matrix Assisted Laser Desorption Ionization mass spectroscopy at the CCIC (Campus Chemical Instrumentation Center, The Ohio State University, Columbus, OH, USA). All fractions were analyzed on analytical RP-HPLC and characterized by MALDI. RP-HPLC fractions showing same mass spectrum peak were pooled together and lyophilized.

### 2.2. Cell Lines and Inhibitors

The human EC cell line OE19 was obtained from Sigma (St Louis, MO, USA) and was grown in regular DMEM. The human TNBC cell line MDA-MB-231 was purchased from American Type Culture Collection (Manassas, VA, USA) and maintained according to the supplier’s instructions. OE-19 cells express high levels of HER-1 and HER-2, whereas MDA-MB-231 cells express high levels of HER-1 and IGF-1R proteins. All growth media, FBS and other supplements were obtained from Invitrogen. Cetuximab and trastuzumab were purchased from The James Cancer Hospital pharmacy of The Ohio State University Wexner Medical Center (Columbus, OH, USA), and AG825 was purchased from Calbiochem (Billerica, MA, USA).

### 2.3. Rabbits

Peptide vaccine antibodies were raised for each peptide vaccine using pairs of New Zealand white rabbits purchased from Charles River Laboratories (Wilmington, MA, USA) and Harlan Laboratories (Indianapolis, IN, USA). Rabbits were immunized with 1mg of peptide emulsified in Montanide ISA 720 (Seppic, Paris, France) and nor-MDP adjuvant (*N*-acetyl-glucosamine-3 yl-acetyl l-alanyl-d-isoglutamine) and boosted twice as previously described [73]. For all experiments, antibody titers were monitored by direct ELISA against the peptide immunogen and the peptide B cell epitope. Sera was collected weekly (3Y + 3W, final bleed three weeks after the third immunization). Peptide vaccine antibodies were purified by affinity chromatography using a protein A/G column and the concentration was measured by Coomassie protein assay. All experiments were performed in accordance with the U.S. Public Health Service Policy on Humane Care and Use of Laboratory Animals and approved by the Ohio State University Institutional Animals Care and Use Committee and detailed in the accepted protocol.

### 2.4. MTT Cell Growth Proliferation Assay.

The assay was performed using human EC cell line (OE-19) and TNBC cell line (MDA-MB-231). Cells were plated in a 96-well plate, as previously described [74]. After incubation for 24 h, growth media were aspirated and replaced with low serum growth media before growing for another 24 h. The following day, cells were treated with single peptide mimics, a combination of peptide mimics, single peptide antibodies, or a combination of peptide vaccine antibodies. The peptide mimics or peptide vaccine antibodies were specifically against HER-1 and HER-2 for OE19 and against HER-1 and IGF-1R for MDA-MB-231. After treatment, the cells were incubated for one hour before being stimulating with ligands for the targeted receptors at a concentration of 50 ng/mL. Cell were then incubated for three days at which point MTT was added to the plates and incubated for 2 h before adding extraction buffer. The plate was incubated overnight before being read on spectrophotometer at 570 nm. The percentage inhibition was calculated using the following formula OD_UNTREATED_ − OD_TREATED_/OD_UNTREATED_ × 100.

### 2.5. Receptor Phosphorylation Assay

Upon ligand binding, the HER-1 and IGF-1R receptors become activated. Homodimerization or heterodimerization with other HER receptors (HER-2 and HER-3) occurs, leading to tyrosine phosphorylation and intracellular signaling. We used the same human EC and TNBC cell lines described above in the proliferation assay to measure the amount of phosphorylated receptors after treatment with single and combinations of peptide mimics or peptide vaccine antibodies. After treatment, cells were washed with cold PBS and lysed with RIPA buffer. The levels of phosphorylated proteins were measured using Human-phosphor HER-1, HER-2 and IGF-1R Duoset kits (R&D Systems, Minneapolis, MN, USA), as previously described [75,76].

### 2.6. Caspase Activity Assay for Apoptosis

Apoptosis was evaluated by measuring levels of caspase 3/7 activity as previously described [70,75]. EC and TNBC cells were plated in 96-well microtiter plates as described in the proliferation assay and incubated overnight at 37 °C. Low serum growth media containing single or combination of peptide mimics or peptide vaccine antibodies were added to the wells. The plates were then incubated for 2 h at 37 °C. The caspase reagent was then added, and levels of released caspases were measured using an illuminometer to assess apoptosis.

### 2.7. Antibody Dependent Cellular Cytotoxicity (ADCC)

We also evaluated the ability of single and combination treatments of peptide vaccine antibodies to cause ADCC of EC and TNBC cells. Peripheral blood mononuclear cells (PBMCs) from normal human donors were used as effector cells and serially diluted in a 96 well plate. The target cells (EC and TNBC) were treated with 100 μg of single or combination peptide vaccine antibodies and controls at an effector to target ratio of 100:1, 20:1 and 4:1 and incubated at 37 °C for 2–4 h. After treatment, cell lysis was measured using a very sensitive non-radioactive cytotoxicity kit from aCella-TOX (Cell Technology, Inc.; Fremont, CA, USA). The experiment was completed according to manufacturer’s recommendation and as previously described [77].

### 2.8. Western Blotting

Cells were lysed in RIPA buffer (Cell Signaling; Danvers, MA, USA) supplemented with protease and phosphatase inhibitors (Sigma-Aldrich). Total protein extracts were run on SDS-PAGE and blotted onto nitrocellulose. Blots were probed overnight. The following antibodies were purchased from Cell Signaling: Rabbit anti-phospho-IGF-1 receptor β (Tyr1131) (#3021, 1:200); rabbit anti-IGF-1 receptor β (#3018, 1:250); rabbit anti-phospho-p44/42 MAPK (Erk1/2) (Thr202/Tyr204) (#9101, 1:1000); rabbit anti-p44/42 MAPK (Erk1/2) (#9102, 1:1000); rabbit anti-phospho-Akt XP (Ser473) (#9018, 1:1000); rabbit anti-AKT (#9272, 1:1000); rabbit anti-phospho-EGFR (Tyr1148) (#4404, 1:200); and mouse anti-EGFR (#2239, 1:200). Mouse anti-β-actin was purchased from Sigma-Aldrich (AC-15, 1:15,000). All primary antibodies were diluted in 5% BSA/TBS-T. Goat anti-mouse secondary IRDye 800 antibody (#926-32210, 1:10,000) was purchased from Li-Cor Biosciences (Lincoln, NE, USA). Goat anti-rabbit alexa-fluor 680 secondary antibody (#1027681, 1:10,000) was purchased from Invitrogen (Grand Island, NY, USA). Protein bands were detected using the Odyssey Imaging System (Li-Cor Biosciences, Lincoln, NE, USA).

### 2.9. Statistical Analysis

Differences in MTT cell proliferation assay, receptor phosphorylation assay, caspase assay and ADCC were evaluated with the Student’s t test. The significance level was set at α = 0.05 which is within the 95% confidence intervals.

## 3. Results and Discussion

### 3.1. Epitope Selection, Design, Synthesis and Characterization

In this study, we selected five novel B-cell epitopes that have been previously characterized by our laboratory. These include one HER-1 epitope (HER-1-418-435) [70], two HER-2 epitopes (HER-2-266-296 and HER-2-597-626) [68,69] and two IGF-1R epitope (IGF-1R-56-81 and IGF-1R-233-251) [76]. Four HER-1 epitopes were designed based on the ligand binding site of the receptor using crystallographic structures, mutagenesis studies and models receptor-ligand interactions [78]. Selection of the best HER-1 epitope (sequence 418–435) was based on its (i) overall *in vitro* antitumor properties: inhibition of cancer cell growth, prevention of HER-1-specific phosphorylation, down regulation of HER-1 signaling pathways, and increased apoptosis and ADCC in HER-1-expressing cells [70]; and (ii) both vaccination with the chimeric MVF-HER-1-418-435 peptide (* *p* < 0.05) and/or treatment with the HER-1-418-435 peptide mimic (* *p* < 0.005) delayed tumor growth in the FVB/n Met-1 transplantable breast cancer model. Additionally, there were significant reductions in the number of actively dividing cells and microvascular density as assessed by immunohistochemical staining of tumor sections for actively dividing cells (Ki-67) and blood vessels (CD31). HER-2-266-296 and HER-2-597-626 were respectively designed from the pertuzumab and trastuzumab binding sites of the HER-2 extracellular domain; after synthesis, the peptides were characterized as previously described [68,69]. Both HER-2 constructs elicited high-affinity peptide vaccine antibodies that inhibited multiple signaling pathways including HER-2/neu-specific inhibition of cellular proliferation and cytoplasmic receptor domain phosphorylation, and caused ADCC. The 266-296 peptide vaccine significantly reduced tumor onset in both transplantable tumor models (FVB/n and BALB/c) and significantly reduced in tumor development in two transgenic mouse tumor models (BALB-neuT and VEGF(+/−)Neu2-5(+/−)). The 597-626 epitope significantly reduced tumor burden in transgenic BALB-neuT mice. We also identified the IGF-1R-56-81 epitope, which was derived from the IGF-1R ligand-binding domain, by predicting the antigenicity and immunogenicity profiles using computer algorithms, followed by analysis of three dimensional structure of IGF-1R to further delineate the exact epitope based on its secondary structure. The IGF-1R peptide antibodies and peptide mimics inhibited cell proliferation and receptor phosphorylation, induced apoptosis and antibody-dependent cellular cytotoxicity (ADCC), and significantly inhibited tumor growth in the transplantable BxPC-3 pancreatic and JIMT-1 breast cancer models. We found additive antitumor effects for the combination treatment of the IGF-1R 56-81 epitope with HER-1-418-435 and HER-2-597-626 epitopes. Treatment with the IGF-1R/HER-1 or IGF-1R/HER-2 combination inhibited proliferation, invasion, and receptor phosphorylation, and induced apoptosis and ADCC, to a greater degree than single agents [76]. The sequences of all the peptide mimics used in this study and their molecular weights are shown in Table 1.

### 3.2. Single and Combination Treatment with Peptide Mimics or Peptide Vaccine Antibodies of HER-1 and HER-2 in EC or HER-1 and IGF-1R in TNBC Inhibits Proliferation of Cells in an MTT Assay

**Figure 1:**
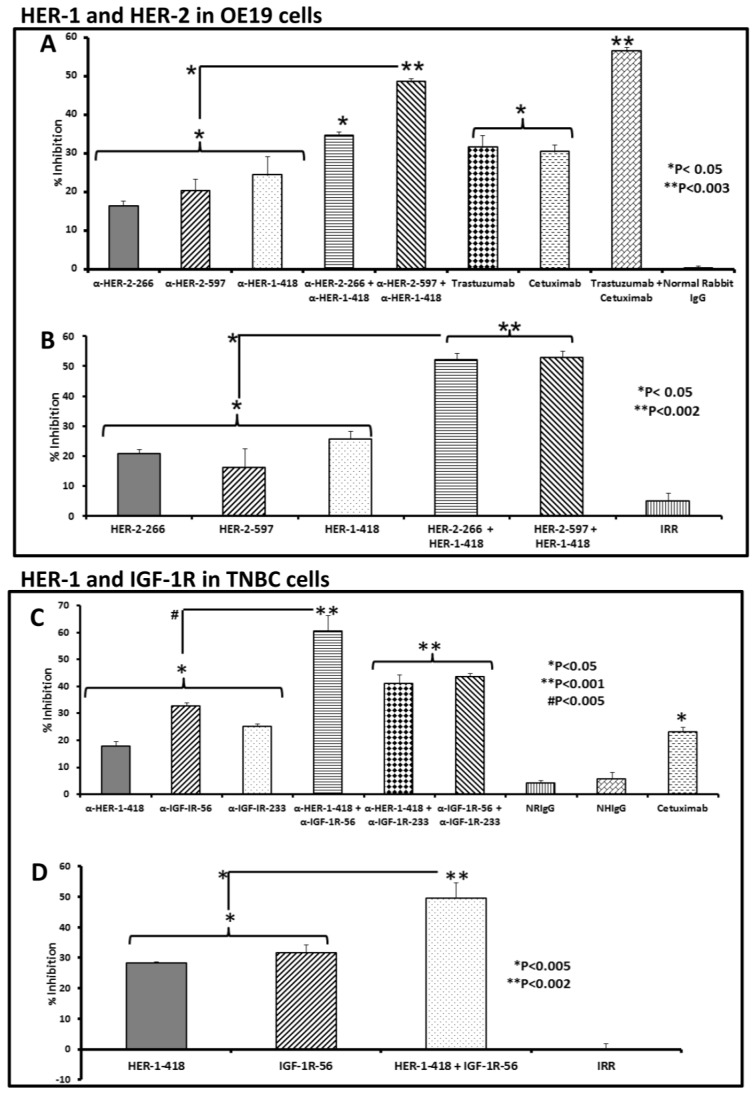
Effects of co-targeting HER-1 and HER-2 in EC or HER-1 and IGF-1R in TNBC. MTT proliferation assay shows significant inhibition of OE19 cells following single and combination treatment with HER-1-418 and HER-2-266 or HER-2-597 peptide vaccine antibodies (**A**) or peptide mimics (**B**) and significant inhibition of TNBC cells following single and combination treatment with HER-1-418 and IGF-1R peptide vaccine antibodies (**C***)* or peptide mimcs (**D**). Cells were treated with peptide mimics or peptide vaccine antibodies for 1 h prior to ligand stimulation with EGF/HRG (50 ng/mL). After 72 h of incubation in the presence of the peptide mimics, MTT was used to measure cell proliferation. Percent inhibition was calculated by taking absorbance (abs) readings at 570 nm and using the following equation: (abs. untreated-abs. treated)/abs. untreated × 100). An irrelevant (IRR) peptide or normal rabbit IgG was used as a negative control; trastuzumab and cetuximab were positive controls. Values represent the mean of at least two independent experiments performed in triplicate (*n* = 3); error bars indicate SD of the mean. Single treatment with either peptide vaccine antibodies or peptide mimics in EC significantly inhibited proliferation over negative control (* *p* < 0.05). The anti-HER-2-597 and anti-HER-1-418 combination (A) also showed significantly higher inhibition compared to single treatment alone (* *p* < 0.05) and combination treatment with both HER-2-266 and HER-1-418 and HER-2-597 and HER-1-418 peptide mimics (**B**) inhibited proliferation more than single treatment alone (* *p* < 0.05). Combination treatment in TNBC cells with anti-HER-1-418 and anti-IGF-1R-56 (**C**) showed greater inhibition of cell proliferation over single treatment alone (# *p* < 0.005) as compared to the anti-HER-1-418 with anti-IGF-1R-233 and anti-IGF-1R-56 with anti-IGF-1R-233 combinations, which significantly inhibited cell proliferation over negative control (**p < 0.001) but did not show a significant advantage over single treatment alone. Similarly, the HER-1-418 and IGF-1R-56 peptide mimics (**D**) showed that the combination treatment inhibited proliferation of TNBC cells significantly more that single treatment alone (* *p* < 0.005).

We evaluated the effects of treatment with the peptide mimics and peptide vaccine antibodies as inhibitors on proliferation of human EC cells (OE19) and TNBC (MDA-MB-231) cells. Ligand binding to HER-1 results in receptor heterodimerization both with itself and with other receptors, including IGF-1R and with the constitutively active HER-2. This heterodimerization activates an intracellular signaling cascade that causes increased cell proliferation. We therefore sought to block receptor heterodimerization using rationally designed peptides or vaccine antibodies and hypothesized that such interventions would decrease *in vitro* proliferation of OE19 and MDA-MB-231 cancer cell lines. We treated the cells with different combinations of peptide mimics or peptide vaccine antibodies as shown in Figure 1, and measured proliferation using an MTT assay. Commercially available drugs trastuzumab and cetuximab were used as a positive controls and rabbit and human IgG was used as negative control in the peptide vaccine antibody experiment. An irrelevant peptide was used as a negative control for the peptide mimic experiment.

**Table 1:** Epitope Selection: Sequences of the peptide mimics used in this manuscript are shown above. In this study, we used five novel B cell epitopes that have been previously characterized in our laboratory and include one HER-1 epitope (HER-1-418-435), two HER-2 epitopes (HER-2-266-296 and HER-2-597-626), and two IGF-1R epitopes (IGF-1R-56-81 and IGF-1R-233-251). These epitopes were designed based on the ligand binding site of the receptor using crystallographic structures, mutagenesis studies, and models of the complex between the receptor ligand interactions. Sequences are also shown for the chimeric B-cell epitope vaccines engineered with the “promiscuous” T cell epitope of the measles virus fusion protein (MVF; italics). The flexible linker sequence GPSL was used in collinearly synthesizing the vaccines with MVF and is underlined in the table; molecular weights for each inhibitor are indicated.

Peptides	Amino Acid Sequence of HER-1,HER-2 and IGF-1R Peptides	Mol.Wt (Da)
HER-1 (418–435)	Ac-SLNITSLGLRSLKEISDG-OH	1944
HER-2 (266–296)	LHCPALVTYNTDTFESMPNPEGRYTFGASCV-OH	3420
HER-2 (597–626)	VARCPSGVKPDLSYMPIWKFPDEEGACQPL-OH	3333
IGF-1R (56–81)	Ac-LLFRVAGLESLGDLFPNLTVIRGWKL-NH_2_	2969
IGF-1R (233–251)	Ac-ACPPNTYRFEGWRCVDRDF-NH_2_	2372
MVF-HER-1 (418–435)	KLLSLIKGVIVHRLEGVE-GPSL-SLNITSLGLRSLKEISDG-OH	4242
MVF-HER-2 (266–296)	KLLSLIKGVIVHRLEGVE-GPSL-LHCPALVTYNTDTFESMPNPEGRYTFGASCV-OH	5757
MVF-HER-2 (597–626)	KLLSLIKGVIVHRLEGVE-GPSL-VARCPSGVKPDLSYMPIWKFPDEEGACQPL-OH	5672
MVF-IGF-1R (56–81)	KLLSLIKGVIVHRLEGVE-LSPG-LLFRVAGLESLGDLFPNLTVIRGWKL-NH_2_	5267
MVF-IGF-1R (233–251)	Ac-KLLSLIKGVIVHRLEGVE-GPSL-Ac-ACPPNTYRFEGWRCVDRDF-NH_2_	4712

Our results in EC cells demonstrated significant dose-dependent inhibition (data not shown) of cell proliferation when cells were treated with HER-1 plus HER-2 peptide vaccine antibodies (Figure 1A), as compared to normal rabbit IgG negative control (* *p* < 0.05). Notably, combination treatment with both anti-HER-1-418 and anti-HER-2-597 significantly inhibited proliferation over single treatment alone (* *p* < 0.05). Similar results were obtained in OE19 cells treated with HER-1 and HER-2 peptide mimics (Figure 1B). Combination treatments of HER-1-418 or HER-2-266 and HER-1-418 and HER-2-597 inhibited proliferation more than single treatment alone (* *p* < 0.05).

The commercially available drugs cetuximab and trastuzumab were used as positive controls and showed higher levels of inhibition than that of the negative control, both individually and in combination (* *p* < 0.05 and ** *p* < 0.003, respectively). Additionally, we used these assays to investigate the effectiveness of two different IGF-1R epitopes (IGF-1R-56 and IGF-1R-233) together or in combination with anti-HER-1-418 in the TNBC cell line. Combination treatment in TNBC cells with anti-HER-1-418 and anti-IGF-1R-56 showed greater inhibition of proliferation over single treatment alone (# *p* < 0.005) as compared to the anti-HER-1-418 and anti-IGF-1R-233 or anti-IGF-1R-56 and anti-IGF-1R-233 combinations, which significantly inhibited cell proliferation over negative control (** *p* < 0.001), but did not show an increased advantage over single treatment alone (Figure 1C). Similar assays with the HER-1-418 and IGF-1R-56 peptide mimics showed supporting results, with combination treatment inhibiting proliferation significantly more that single treatment alone (* *p* < 0.005). Single treatment alone also conferred a decrease in cell proliferation, indicating a potential benefit of co-targeting HER-1 and IGF-1R in TNBC (Figure 1D).

Taken together, these data show that combination treatment, with peptide mimics or peptide vaccine antibodies, significantly decreased cell proliferation, indicating the potential benefits of targeting both HER-1 and HER-2 in EC or HER-1 and IGF-1R in TNBC. Importantly, both the peptide vaccine antibodies and the peptide mimics demonstrated similar mechanisms of action.

### 3.3. Peptide Vaccine Antibodies Induce ADCC of OE19 and TNBC Cells in Vitro

**Figure 2:**
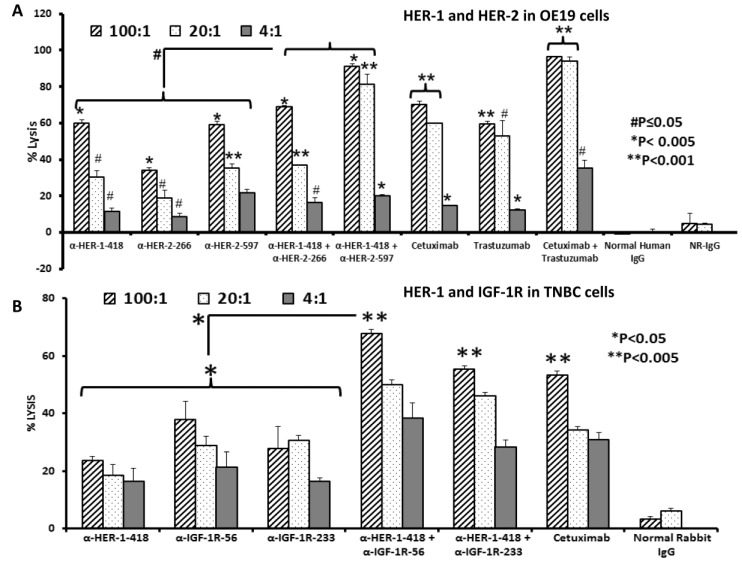
Co-targeting HER-2 and HER-1 or IGF-1R and HER-1 causes greater induction of ADCC in EC and TNBC. OE19 or MDA-MB-231 cells were used as target cells and were seeded and incubated in the presence of human PBMCs at different effector: Target cell ratios (100:1, 20:1, 4:1). Cells were then treated for one hour with peptide vaccine antibodies prior to cell lysis. The aCella-tox kit (Cell Technology, Inc.; Fremont, CA, USA) was used to measure the relative amount of ADCC; cell lysis was measured according to the manufacturer’s instructions. Results represent an average of two different experiments performed in triplicate and display the % lysis of treatment groups when compared to 100% target cell lysis. Normal human and rabbit IgG (Pierce, Rockford, IL, USA) were used as a negative control; cetuximab and trastuzumab were positive controls. In EC cells, at a 100:1 ratio (**A**), single treatment with the anti-HER-2 and anti-HER-1 peptide vaccine antibodies showed significant induction of ADCC (* *p* < 0.005), but combination treatment using the anti-HER-1-418 and anti-HER-2-597 or anti-HER-1-418 and anti-HER-2-266 peptide vaccine antibodies caused greater induction of ADCC (# *p* < 0.05) than single treatment alone. In TNBC cells, at a ratio of 100:1 (**B**), anti-HER-1-418, anti-IGF-1R-56, and anti-IGF-1R-233 peptide vaccine antibodies induced significant levels of ADCC as compared to negative control (* *p* < 0.05). The combination of anti-HER-1-418 and anti-IGF-1R-56 induced significantly higher levels of ADCC compared to single treatment alone (* *p* < 0.05).

Active immunization with the MVF-HER-1, MVF-HER-2 and MVF-IGF-1R chimeric peptides elicited the production of high-affinity antibodies that induced ADCC. ADCC is a major mechanism of action used by antibodies in cell defense. In this process, Fc regions interact with receptors on PBMCs and attract them to specific cellular targets. We performed a non-radioactive ADCC assay to determine whether HER-1 and HER-2 vaccine antibodies can induce ADCC using effector PBMCs from normal human donors and OE-19 cells target cells as previously described [77]. Maximum lysis was measured using the aCella-TOX reagent kit from Cell Technology, Inc. (Fremont, CA, USA). HER-2 and HER-1 peptide vaccine antibodies in combination and alone induced significantly higher levels of ADCC as compared to negative control (Figure 2A, (# *p* ≤ 0.05, * *p* < 0.005, ** *p* < 0.001)). At an effector: Target cell ratio of 1:100, both combinations of HER-1-418 and HER-2-597 or HER-1-418 and HER-2-266 induced significantly higher levels of ADCC than single treatment alone (# *p* ≤ 0.05). These results provide additional evidence supporting the potential benefits of targeting HER-1 and HER-2 as novel combinations in EC expressing these receptors.

In TNBC cells, we again examined two different IGF-1R epitopes (IGF-1R-56 and IGF-1R-233) alone or in combination with anti-HER-1-418. Anti-HER-1-418 and anti-IGF-1R-56 peptide vaccine antibodies, in combination and alone, induced significantly higher levels of ADCC as compared to negative control (* *p* < 0.05, ** *p* < 0.005) (Figure 2B). Notably, the combination of anti-HER-1-418 and anti-IGF-1R-56 uniquely induced significantly higher levels of ADCC compared to single treatment alone (* *p* < 0.05). The enhanced effects of the HER-1-418 and IGF-1R-56 combinations informed our selection of the IGF-1R-56 epitope for the following experiments in TNBC and in sum, these results provide additional supporting evidence for the potential benefits of targeting multiple receptors as novel combinations in EC and TNBC.

### 3.4. Single and Combination Treatment with Peptide Mimics or Peptide Vaccine Antibodies of HER-1 and HER-2 in EC or HER-1 and IGF-1R in TNBC down Regulates Receptor Phosphorylation in Vitro

**Figure 3:**
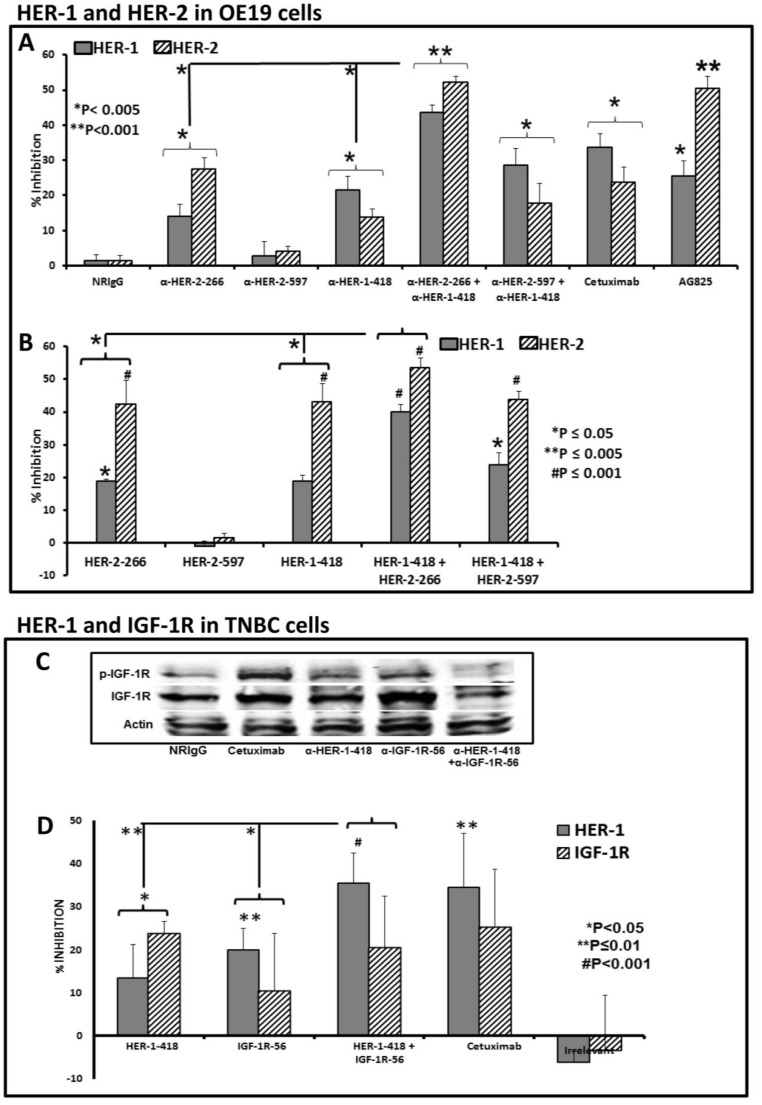
Effects of co-targeting HER-1 and HER-2 or HER-1 and IGF-1R on receptor phosphorylation in EC or TNBC. OE19 or MDA-MB-231 cells were treated for 1 h prior to ligand stimulation with 10 ng/mL EGF/HRG for 15 min. After treatment, cells were lysed 1× in RIPA lysis buffer (Santa Cruz Biotechnology, Inc; Dallas, TX, USA). Phosphorylated HER-1, HER-2, and IGF-1R were measured via western blot or by phospho-ELISA kits from R&D Systems. Percent inhibition was calculated by taking absorbance (abs) readings at 450 nm and using the following equation: (abs. untreated-abs. treated)/abs. untreated × 100). Results displayed are representative of two independent experiments performed in triplicate. Error bars represent SD of the mean. Single peptide vaccine antibody treatments with anti-HER-1-418 or with anti-HER-2-266 (**A**) significantly decreased phosphorylation of both receptors as compared to negative control (* *p* < 0.005). The combination of anti-HER-2-266 and anti-HER-1-418 in OE19 cells showed significant down-regulation of receptor phosphorylation over single treatment alone (* *p* < 0.005). The peptide mimic combination of HER-1-418 and HER-2-266 (**B**) inhibited phosphorylation significantly more than single treatment alone (* *p* ≤ 0.05) with the exception of the comparison to the HER-2 receptor in the HER-1-418 single condition (*p* = 0.09). Western blots of total protein lysates were performed for p-Tyr1131 IGF-IR (1:200), total IGF-IR (1:250) (both from Cell Signaling), and beta-actin (1:15,000) (Sigma-Aldrich). Goat anti-mouse secondary IRDye 800 antibody (#926-32210, 1:10,000) was purchased from Li-Cor Biosciences (Lincoln, NE, USA) and goat anti-rabbit alexa-fluor 680 secondary antibody (#1027681, 1:10,000) was purchased from Invitrogen (Grand Island, NY, USA). Protein bands were detected using the Odyssey Imaging System (Li-Cor Biosciences, Lincoln, NE, USA). Results with peptide vaccine antibodies in TNBC cells (**C**) showed increased inhibition of receptor phosphorylation with combination treatment, as demonstrated by western blot analysis, where treatment with anti-HER-1-418 and anti-IGF-1R-56 together markedly decreased IGF-1R phosphorylation as compared to control (normal rabbit IgG) or single peptide vaccine antibody treatment alone. Single peptide mimic treatment (**D**) with HER-1-418 decreases phosphorylation of both HER-1 and IGF-1R receptors significantly over negative control (* *p* < 0.05), while single treatment with IGF-1R-56 significantly inhibits phosphorylation of the HER-1 receptor (** *p* < 0.01), as determined by phospho-ELISA analysis. Combination treatment with IGF-1R-56 and HER-1-418 showed significant down-regulation of both receptor phosphorylation over single treatment with IGF-1R or HER-1-418 alone (* *p* < 0.05, ** *p* < 0.01 respectively).

Next, we evaluated the effects of the peptide mimics and peptide vaccine antibodies on HER-1 and HER-2 receptor phosphorylation in OE19 cells. Signaling through these receptors activates downstream pathways, including MAPK and AKT, and increases expression and phosphorylation of the receptors. Phosphorylated levels of HER-1 and HER-2 were measured with a sandwich ELISA method with the human-phospho-HER-1 or HER-2 ELISA kit from R&D systems. In Figure 3, we show that both peptide mimics and peptide vaccine antibodies down-regulate phosphorylation of HER-1 and HER-2 in OE19 cancer cells *in vitro*.

Specifically, in EC cells (OE19), single peptide vaccine antibody treatment decreases phosphorylation significantly over negative control (normal rabbit IgG) (* *p* < 0.005) (Figure 3A). Combination treatment with anti-HER-2-266 and anti-HER-1-418 showed significant downregulation of receptor phosphorylation over single treatment alone (* *p* < 0.005). In this assay, the combination of anti-HER-2-266 with anti-HER-1-418 appears to confer advantage over the anti-HER-2-597 combination. The anti-HER-2-597 trastuzumab-like epitope did not significantly inhibit phosphorylation. This result is not surprising, as trastuzumab binds to sub domain III of HER-2 but does not interfere with dimerization in domain II; therefore, there should be no effect on HER-2 phosphorylation. The combination HER-1-418 and HER-2-266 peptide mimics inhibited phosphorylation significantly more than single treatment alone (* *p* < 0.05) although inhibition of phosphorylation of the HER-2 receptor differed with the HER-1-418 single condition (*p* = 0.09) (Figure 3B). Most importantly, the best inhibition was obtained following combination treatment with HER-2 266 and HER-1-418 peptide mimics or peptide vaccine antibodies, validating this combination as a potentially beneficial strategy for EC.

Treatment with peptide vaccine antibodies in TNBC cells (MDA-MB-231) showed increased inhibition of receptor phosphorylation with the combination treatment of anti-HER-1-418 and anti-IGF-1R-56. Results were determined by western blot analysis of total protein lysates performed for p-Tyr1131, IGF-IR, total IGF-IR, and beta-actin. Combination treatment with peptide vaccine antibodies shows a marked decrease of IGF-1R phosphorylation as compared to control (normal rabbit IgG) or single peptide antibody treatment alone (Figure 3C). Furthermore, single peptide mimic treatment decreased phosphorylation significantly over negative control (irrelevant peptide) (* *p* < 0.05) and combination treatment with IGF-1R-56 and HER-1-418 showed significant down-regulation of receptor phosphorylation over single treatment with IGF-1R or HER-1-418 alone (* *p* < 0.05, **p < 0.01 respectively) (Figure 3D).

### 3.5. Apoptosis Determination of OE19 and MDA-MB-231 Cancer Cells in Vitro by Caspase Activity Assay

**Figure 4:**
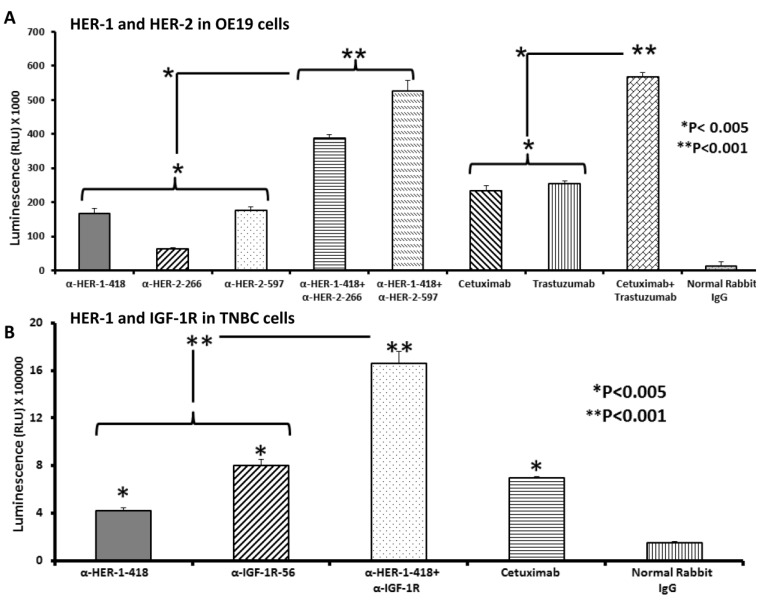
Effects of co-targeting HER-1 and HER-2 or HER-1 and IGF-1R on induction of apoptosis in EC or TNBC. OE19 or MDA-MB-231 cancer cells were plated in 96-well plates and treated with peptide vaccine antibodies for 24 h prior to cell lysis. Apoptosis (directly proportional to amount of luminescence produced) was measured using the Caspase Glo 3/7 kit (Promega, Madison, WI, USA). After 24 h of treatment, caspase glo reagent was added, and plates were incubated for 3 h before being read on a luminometer. Normal rabbit IgG was used as a negative control; cetuximab and trastuzumab were positive controls. Results are representative of two independent experiments performed in triplicate. Error bars represent SD of the mean. In EC cells (**A**), single peptide vaccine antibody treatment significantly increased apoptosis over negative control (* *p* < 0.005). The combinations of anti-HER-1-418 and HER-2-266 or anti-HER-1-418 and HER-2-597 showed significantly higher levels of apoptosis than single treatment alone (* *p* < 0.005). TNBC cells (***B***) showed single peptide vaccine antibody treatment significantly increased apoptosis over negative control (* *p* < 0.005), and the combination of anti-HER-1-418 and anti-IGF-1R-56 showed significantly higher levels of apoptosis compared to single treatment alone (** *p* < 0.001).

In Figure 4, we evaluated the induction of apoptosis following *in vitro* combination treatment with HER-1 and HER-2 or HER-1 and IGF-1R peptide vaccine antibodies using a caspase activity assay with EC (OE19) or TNBC (MDA-MB-231) cells. Briefly, cells were plated on a 96-well, non-transparent plate and treated with single or combination treatments of peptide vaccine antibodies. Trastuzumab and cetuximab were used as positive controls, and normal rabbit IgG was used as a negative control. Caspase activity was determined by measuring levels of caspase 3 and 7 using the caspase-Glo reagent, critical enzymes that are released during apoptotic programmed cell death.

Single peptide vaccine antibody treatment in EC cells significantly increased apoptosis over negative control (* *p* < 0.005). The combinations of anti-HER-1-418 with HER-2-266 and anti-HER-1-418 with HER-2-597 showed significantly higher levels of apoptosis than single treatment alone (* *p* < 0.005), in addition to higher levels than negative control (**p < 0.001) (Figure 4A). The combination of cetuximab and trastuzumab also increased apoptosis significantly over single treatment of either commercial drug alone (* *p* < 0.005). In TNBC cells, while single peptide vaccine antibody treatment significantly increased apoptosis over negative control (* *p* < 0.005), the combinations of anti-HER-1-418 with anti-IGF-1R-56 showed significantly higher levels of apoptosis over single treatment alone (** *p* < 0.001), in addition to higher levels than negative control (** *p* < 0.001) (Figure 4B). Combined, these results further highlight the potential therapeutic benefits of targeting both HER-1 and HER-2 in EC or HER-1 and IGF-1R in TNBC.

## 4. Discussion

The studies described in this paper revolve around the central goal of developing novel immunotherapeutic combinations of peptide-based therapeutics and vaccines to provide safer, less toxic alternatives and to overcome mechanisms of resistance. Current FDA-approved treatment regimens, such as humanized monoclonal antibodies and small molecule inhibitors, exhibit significant toxicity, problems with selectivity and efficacy, and excessively high rates of acquired resistance (up to 70% within a year). Given that there is no valid mouse model for active immunization for EC and TNBC with peptide vaccines, the studies reported here are predicated on the following assumptions: (i) the development of peptide vaccine antibodies that can be used as surrogates in xenografts models to assess efficacy of our peptide vaccine; (ii) the peptide mimic approach is complementary to the peptide vaccine antibodies strategy to corroborate and validate the epitope discovery strategy; (iii) finally, the peptide mimic can be used in a safe therapeutic setting. We hypothesized that rational combinations of peptide mimics or peptide vaccine antibodies targeting multiple receptor tyrosine kinases would provide additive and/or synergistic inhibition of tumor growth and suppress metastasis of multiple cancer types.

In the present study, we focused on two diseases, namely EC and TNBC, because highly effective targeted therapies have yet to be established for these tumor types. EC has a very high fatality rate and is one of the fastest rising cancers worldwide [79]. EC patients in which HER-2 is overexpressed, normally treated with trastuzumab in combination with cytotoxic chemotherapy have only modest survival gains and suffer from intrinsic or acquired resistance. TNBCs represent a significant treatment challenge, as they have a relatively poor prognosis because it is evidently a heterogeneous disease comprising multiple redundancies and overlapping signaling pathways cross-talk. Thus, in both cases the efficacy of a one-dimensional therapeutic strategy in which only one pathway is inhibited would eventually be undermined by the upregulation and activation of a compensatory pathway. By contrast, combining two or more targeted agents may provide a more effective approach for treating EC or TNBC.

The significance of our approach is that rationally designed peptide vaccines/mimics represent a viable therapeutic strategy for blocking aberrant molecular signaling pathways with high affinity, high specificity, strong potency and improved safety profiles. The work presented in this manuscript addresses this need by developing and evaluating two therapeutic combination strategies utilizing novel peptide mimics and peptide vaccine antibodies. The overall mechanism of anti-tumor action involves the binding of the antibody or the peptide mimic to the receptor target on the tumor cell, blocking signal transduction (inhibiting receptor homodimerization or heterodimerization) and eliciting a variety of anti-tumor cellular mechanisms e.g., ADCC (in the case of antibodies), inhibition of proliferation or phosphorylation, apoptosis and tumor cell death. In this study, we have evaluated the *in vitro* efficacy of combination therapy targeting HER-1 and HER-2 in EC cells (OE19) and HER-1 and IGF-1R in TNBC cells (MDA-MB-231).

We aimed to develop strategies that could circumvent resistance mechanisms in EC by dually targeting two receptors (HER-1 and HER-2) implicated in this cancer. In addition to evaluating combination therapy, these experiments tested the efficacy of our novel peptide mimics or peptide vaccine antibodies on esophageal tumorigenesis. Recent work has shown that HER-2 overexpressing tumors exhibit upregulation of another gene, resulting in secondary oncogenic effects (cell cycle control, increased kinase signaling), which likely contribute to resistance to mono targeted therapy [80]. Moreover, when these secondary oncogenes (such as HER-1) are targeted in conjunction with HER-2 therapy, the *in vitro* anti proliferative effects of single treatment are augmented, supporting the importance of combination therapies in novel cancer treatment. Co-treatment with trastuzumab and cetuximab has shown promising anti proliferative effects *in vitro* in a human non-small cell lung carcinoma cell line (A549) [81].

In our *in vitro* experiments in EC human cells (OE19), the combination of HER-1-418 (cetuximab-like) and HER-2-266 (pertuzumab-like) or HER-2-597 (trastuzumab-like) peptide mimics or peptide vaccine antibodies demonstrated superior anti-tumor responses over single treatment. Combination therapy with peptide vaccine antibodies and peptide mimics inhibited tumor growth by direct mechanisms, including decreased cell proliferation and receptor phosphorylation to a greater degree than individual treatment. Furthermore, anti-HER-1-418 in combination with anti-HER-2-266 or anti-HER-2-597 vaccine antibodies also induced greater levels of ADCC and apoptosis than single treatment, exhibiting indirect methods of tumor suppression as well. Interestingly, depending on the mechanisms being measured, the HER-2-597 or HER-2-266 epitope had greater effects in combination with HER-1-418 treatments. The HER-2-266 and HER-2-597 epitopes were created to mimic  pertuzumab and trastuzumab, respectively, and thus, exert their anti-tumor effects in different ways. While HER-2-266 interacts with the center of domain II and likely directly interferes with receptor dimerization, the HER-2-597 trastuzumab like epitope binds the c-terminal of the extracellular region which blocks activation of HER-2 by promoting receptor endocytosis as well as blocking proteolytic cleavage of the ECD [82]. The individual effects of either HER-2-597 or HER-2-266 are no better alone in any of the *in vitro* measurements, but their unique added strength with HER-1-418 in the *in vitro* tumorigenesis of OE19 EC cells suggests a potential treatment advantage of specific combinations in future *in vivo* experiments.

Our second goal was to study the efficacy of targeting both HER-1 and IGF-1R in TNBC cell lines. HER-1 expression and pathway activation are common in TNBC; however, anti-HER-1 therapies have not been effective in this patient population. A number of HER-1 inhibitors have been extensively investigated as both monotherapies or in combination with other treatments. There is a wealth of evidence that implicates the insulin-like growth factor-1 receptor (IGF-1R) as a major target in cancer drug discovery and its role in the development of resistance to targeted therapies. Crosstalk between IGF-1R and HER-1 has also been extensively described in TNBC [58].

The combination of HER-1-418 and IGF-1R-56 peptide mimics on TNBC cells (MDA-MB-231) *in vitro* demonstrated superior anti-tumor efficacy over single treatment. Combination therapy with peptide vaccine antibodies and peptide mimics inhibited cell proliferation and decreased receptor phosphorylation to a greater degree than treatment with one or the other on its own. Furthermore, anti-HER-1-418 and anti-IGF-1R-56 vaccine antibodies induced greater levels of ADCC and apoptosis than single treatment. Our *in vitro* results of dual inhibition of HER-1 and IGF-1R point to new potentials for clinical treatment for TNBC. IGF-1R is a critical component in this combination therapy, as it is overexpressed in a variety of malignant cancers and is hypothesized to contribute significantly to the development of resistance to HER-1 (cetuximab) targeted therapies [83,84,85] due to involvement in numerous cell signaling cascades [22,86].

## 5. Conclusions

In conclusion, we report here the combination of HER-1 and HER-2 or HER-1 and IGF-1R peptide mimics capable of inducing vaccine antibodies with antitumor properties that significantly reduce measures of tumorigenesis in *in vitro* models of EC and TNBC. The results presented provide a mechanistic understanding of how HER-1/IGF-1R and HER-1/HER-2 signaling influences complex biological processes in these cancer cell lines, and the promising results support the rationale for dual targeting with HER-1 and HER-2 or IGF-1R as an improved treatment regimen for advanced therapy tailored to EC and TNBC. Finally, our data suggests that combination therapy with peptide mimics or peptide vaccines may offer a more effective and safer treatment option than current monoclonal antibody such as trastuzumab or cetuximab. Current monoclonal antibody therapies are limited by complications, including unequal tissue distribution, limited half-life, prolonged administration, possible immunogenicity with high dosages, and cardiotoxicity. Additionally, peptide mimic and peptide vaccine production is simple, reliable, and cost effective. Going forward, we will test the validity of these two different combinations *in vivo* in transplantable mouse models as the ultimate test of preclinical efficacy prior to potential human clinical trials.
